# Examination of intestinal ultrastructure, bowel wall apoptosis and tight junctions in the early phase of sepsis

**DOI:** 10.1038/s41598-020-68109-9

**Published:** 2020-07-13

**Authors:** Beate Obermüller, Noemi Frisina, Martin Meischel, Georg Singer, Stefanie Stanzl-Tschegg, Helga Lichtenegger, Dagmar Kolb, Ingeborg Klymiuk, Holger Till, Christoph Castellani

**Affiliations:** 1 Department of Biomedical Research, Medical University of Graz, Graz, Austria; 2 Institute of Physics and Materials Science, University of Natural Resources and Life Sciences, Vienna, Austria; 3 Department of Paediatric and Adolescent Surgery, Medical University of Graz, Graz, Austria; 4 Core Facility Ultrastructure Analysis, Center for Medical Research, Gottfried Schatz Research Center, Medical University of Graz, Graz, Austria; 5 Core Facility Molecular Biology, Center for Medical Research, Medical University of Graz, Graz, Austria; 6 Division of Cell Biology, Histology and Embryology, Gottfried Schatz Research Center, Medical University of Graz, Graz, Austria

**Keywords:** Bacterial infection, Experimental models of disease

## Abstract

Gut hyperpermeability can be caused by either apoptosis of the intestinal epithelium or altered status, permeability or porosity of tight junctions. This project aims to elucidate these mechanisms in the early phase of sepsis. Eighteen male wild type mice were randomized to two groups. All mice received one single gavage of fluorescein isothiocyanate (FITC) dextran 30 min before intervention. One group (n = 10) underwent cecal ligation and puncture to induce sepsis. The other group (n = 8) was sham operated. Septic animals exhibited significantly increased permeability for FITC 8 h post-operatively. Significantly increased serum interleukin-6, tumor-necrosis-factor-alpha and interleukin-1-beta confirmed sepsis. Septic animals showed significant bowel wall inflammation of ileum and colon samples. PCR revealed significantly increased expression of claudin-2 and decreased expressions of claudin-4, tight-junction-protein-1 and occludin-1 resembling increased permeability of tight junctions. However, these alterations could not be confirmed at the protein level. Light microscopy revealed significant dilatation of intercellular spaces at the basal sections of intestinal epithelial cells (IEC) in septic animals confirmed by increased intercellular spaces at the level of tight junctions and adherens junctions in electron microscopy (TEM). In small angle X-ray scattering no increase in number or size of nanopores could be shown in the bowel wall. HOECHST staining and PCR of ileum samples for apoptosis markers proofed no relevant differences in intestinal epithelial cell apoptosis between the groups. Intestinal hyperpermeability in septic animals was most likely caused by alterations of the intercellular contacts and not by apoptosis or increased size/number of nanopores of intestinal epithelial cells in this murine model of early sepsis.

## Introduction

Sepsis still represents a significant worldwide health care problem despite recent advances in intensive care medicine. In the United States, more than 800,000 patients develop sepsis and between 230,000 and 370,000 patients die from this disease annually^1^.

The bowel wall represents one of the most important functional barriers of the human body. The intestinal epithelial cells (IECs) form its innermost layer and play a major role in intestinal barrier function^2^. This barrier works as a selective filter allowing trans- and paracellular transport of nutrients, electrolytes and liquids while blocking bacteria and their toxins from entering the circulation^3,4^. Disruptions of the barrier function have been reported in sepsis, but also a variety of other diseases such as chronic inflammatory bowel disease, type 2 diabetes and tumor associated cachexia^5^. An increased permeability in these cases can be caused by either structural alterations of tight junction components or by apoptosis of IECs^6^. Additionally, ultrastructural alterations of the IEC architecture can be hypothesized. Many research projects in the past have focused on the investigation of the gut barrier applying various methods. Amongst others, the permeability for fluorescein isothiocyanate dextran (FITC-dextran), electron microscopy, conventional histology, immunohistochemistry and quantitative analysis of tight junction components have been described^7,8^. However, none of these techniques can offer detailed information about the ultrastructure of IECs.

Small-angle X-ray scattering (SAXS) is a non-destructive experimental method used for ultrastructure analysis in materials sciences^9^. A sample is hit by an X-ray beam and while the major part of this beam passes the sample unperturbed, a small proportion is scattered at zones of different densities—for instance the border between lipid bilayers and the cytosol^10^. SAXS has been used to study a variety of biological materials^11–14^ including soft tissues^9,14–16^. However, until now SAXS has not been used to assess nanostructural alterations of the bowel wall during sepsis.

Thus, it was the aim of this experimental study to apply a combination of materials characterization techniques such as SAXS, light and electron microscopy to study the ultrastructure of the bowel wall in the early phase of sepsis. Additionally, we sought to investigate mechanisms (status and composition of tight junctions, epithelial apoptosis or altered cellular architecture) leading to gut hyperpermeability.

## Material and methods

18 male C57Bl/6 mice were obtained from Harlan Laboratories at an age of 7 weeks. After delivery, mice were accustomed to the new surroundings for 1 week. The animals had free access to chow and water at all times and were subjected to a 12 h light–dark cycle. After 1 week the animals were split into two groups by weight. All animals were kept under the same housing conditions and in the same rack. Mice of both groups were gavage fed with 500 mg/kg FITC-dextran (Sigma Aldrich Handels GmbH, Vienna, Austria) dissolved in PBS puffer at 50 mg/ml. Thirty minutes after gavage animals of the sepsis group (n = 10) were anesthetized with 0.05 mg/kg fentanyl, 5 mg/kg midazolam and 0.5 mg/kg meditomedine. Under temperature control mice were shaved and the skin disinfected. Mice received a median laparotomy and cecal ligation and puncture (CLP) was performed. Briefly, the cecum was mobilized, ligated and punctured with a 20 G needle as described before^17^. After wound closure in double layers anesthesia was antagonized with 2.5 mg/kg atipamezole and 0.5 mg/kg flumazenil. Animals of the control group (n = 8) underwent sham operation consisting of general anesthesia, median laparotomy, exteriorization and reduction of the cecum and abdominal closure. Postoperatively, animals of both groups received 0.1 mg/kg subcutaneous buprenorphine as pain therapy. Behavior of the animals was constantly monitored for severe pain or adverse events using a standardized scoring system as required by the governmental veterinary board (Supplement 1).

Eight and a half hours after FITC-dextran gavage mice were re-anesthetized. The heart was punctured with a 23 G needle and blood was harvested. Mice were then sacrificed by cranio-cervical dislocation. The abdomen was opened to dissect 4 cm of terminal ileum (starting 1 cm orally of the ileo-cecal valve) and 4 cm of colon (starting 1 cm caudally of the ileocecal valve).

### Inflammatory cytokines

Pro-inflammatory cytokines in serum were determined with a Millipore Merck Luminex® Kit (MMHMAG-70 K, Merck Chemicals and Life Science GmbH, Vienna, Austria) which was customized for Interleukin (IL)-1α, IL-1β, IL-6, Tumor Necrosis Factor (TNF)-α and Interferon (INF)-γ. Measurements were conducted according to the manufacturer’s instructions.

### FITC-dextran assay for gut permeability

After clotting for 30 min blood was centrifuged for 10 min at 10,000 rpm. 150 μl of the supernatant serum were stored in absolute dark at 4 °C until measurement. The serum FITC-dextran content was determined fluorometrically at wavelengths of 485 nm and 535 nm as described in the manufacturer’s instructions.

### Light microscopy of ileum and colon samples

Ileum and colon samples underwent standard histological work-up with fixation, dehydration, embedding, cutting, rehydration followed by hematoxylin–eosin staining. Histological examination for bowel wall inflammation was conducted from three slices per ileum and colon sample in three different magnifications (100 ×, 200 × and 400 ×). The measurements included the determination of villus height, crypt depth and bowel wall inflammation according to a modified Marsh Oberhuber classification^19^. Additionally, the width of the intercellular spaces was determined with ImageJ.Ink 1.52® in hematoxylin–eosin stains of three ileum samples per mouse with a 100 × oil immersion objective lens (Supplement 2).

### Conventional scanning electron microscopy (CSEM)

Of each animal one piece of ileum and one piece of colon were opened on the anti-mesenteric side. The bowel content was removed by careful rinsing with physiologic saline solution. Samples were then fixed with 5% buffered glutaraldehyde at 4 °C for 2 h. After fixation with 1% osmiumtetroxide samples were dehydrated in an ascending alcohol series. The dehydrated specimens were bathed in 1,1,1,3,3,3-hexamethyldisilazan and gold coated with an Edwards Scancode Six® (Hind High Vacuum Company Private Limited, Bangalore, India) for 2 min. SEM was conducted with a Quanta FEG 250® (Thermo Fisher Scientific) electron microscope using a 20 kV beam. Images were taken with xTm 4.1.7.2095® (Thermo Fisher Scientific). Three images from tilting angles of − 5°, 0° and 5° were obtained at a magnification of 1,000 × and used for 3D reconstruction with MeX 5.1® (Alicona Imaging GmbH, Raaba, Austria). From 125 × 200 μm areas of these 3D reconstructions the surface roughness (R_a_) was calculated using a cut-off wavelength of 30 μm.

### Transmission electron microscopy (TEM)

Similar to SEM, one cm of ileum and one cm of colon were prepared for TEM analysis. Specimens were fixed in 2.5% (wt/vol) glutaraldehyde and 2% (wt/vol) paraformaldehyde in 0.1 M phosphate buffer, pH 7.4, for 2 h, postfixed in 2% (wt/vol) osmium tetroxide for 2 h at room temperature. After dehydration (dehydrated in graded series of ethanol), tissues were infiltrated (ethanol and agar 100 epoxy resin, pure agar 100 epoxy resin) and placed in agar 100 epoxy resin (8 h), transferred into embedding moulds, and polymerised (48 h at 60 °C). Ultrathin sections (70 nm thick) were cut with a UC 7 Ultramicrotome (Leica Microsystems, Vienna, Austria) and stained with lead citrate for 5 min and platin blue for 15 min. Images were taken at 120 kV with a Tecnai G 2 FEI microscope equipped with an ultrascan 1,000 ccd camera (Gatan). The images were examined for widening of the intercellular contacts at the level of the TJ, the adherens junction and the desmosomes. Measurements were conducted with ImageJ.Ink version 1.52® at the widest spot each for at least 10 cell–cell contacts per animal (Supplement 2).

### Small-angle X-ray scattering (SAXS)

For the SAXS measurements bowel samples of ileum and colon were opened and rinsed as described above. Gut samples were vacuum-sealed in Kalle Brat® foil (Ed. Haas Austria GmbH, Traun, Austria) and stored at − 80 °C. SAXS measurements were obtained with a Rigaku S-MAX3000 SAXS System (Rigaku Europe SE, Neu Isenburg, Germany) with a Cu-α target Micro Focus X-ray tube MM-002 + (at a voltage of 45 keV and amperage of 0.88 mA with an energy of 8.05 keV). The X-ray beam was focused through two diffraction-free germanium pinholes resulting in a beam diameter of 420 µm at the sample. In order to preserve the structural integrity of the intestinal wall specimens were mounted on a temperature-controlled stage (HFS350X-CAP; Linkam Scientific Instruments, Tadworth, UK) and kept at a temperature of − 21 °C during the measurements. The detector–sample distance was 1,400 mm allowing for a q-range of 0.01–0.6 A^−1^. The scattering vector q is related to the wavelength λ (λ = 1.54 A) and the scattering angle 2θ.

$$q=\frac{4\pi }{\lambda }\cdot \mathrm{sin}\theta $$

Each measurement took 1,500 s and was carried out in vacuum conditions at a maximum pressure of 10^–2^ mbar to minimize absorption and diffraction by air. For measurements the direct beam was blocked with a 3.8 mm beamstop and the scattering profile was recorded with a TRITON 200 2D multi wire gas-filled X-ray detector (Rigaku Europe SE, Neu Isenburg, Germany) with an active diameter of 200 mm.

Since the bowel samples showed an isotropic scattering pattern indicating no preferred structural orientation in the sample, only the scattering intensity I was determined as a function of q.

For this purpose, the 2D scattering profiles were radially integrated and corrected by subtracting the background signal using SaxsGui 2.15.01® software packages. Scattering curves were then plotted as I(q) in a double logarithmic scale.

Distinct peaks observed at 0.1 A^−1^ and 0.15 A^−1^ were attributed to ice crystals due to blood remnants in the samples and excluded from the evaluation. The interpretation of SAXS data mainly focused on the low q range from 0.01 to 0.05 A^−1^, which is attributed to structures in the size range of 12.5–62.8 nm corresponding to potential alterations of tight junction nanopores. Since all plots showed straight lines in the regarded q range the evaluation was limited to fitting an exponential decay with an exponent describing the fractal dimension as described by Porod’s law (where D represents the scaling factor and α the porod exponent).

$$I\left(q\right)=D\cdot {q}^{-\alpha }$$

This equation was implemented in a Python 2.7 script in order to evaluate the fractal dimensions of all samples.

The slope α of the double logarithmic Porod plot yields information about the fractal dimension of the scattering plot. Thus, a Porod exponent α = 1 is obtained from scattering by rigid rods, while α = 2 represents scattering from a flat (almost 2 dimensional) structure, such as lamellae or platelets, α = 4 characterizes smooth and a slope between 3 and 4 rough surfaces.

### Fluorescence immunohistochemistry for IEC apoptosis

HOECHST staining was performed to determine the ratio of apoptotic intestinal epithelial cells of the ileal sections. Fixation and paraffin embedding was performed according to standard histological procedures. 2.5 μm thick paraffin slices were submersed in Roticlear solution (Carl Roth GmbH + Co. KG, Karlsruhe, Germany) followed by 2 × 100% ethanol, 70% ethanol, 50% ethanol, distilled water and PBS puffer for 5 min each. A HOECHST working solution was prepared by diluting 25 mg original medium (HOECHST B2261-25MG, Lot 017K4122, Sigma Aldrich Handels GmbH, Vienna, Austria) to a concentration of 5 μg/ml with distilled water (the HOECHST solution has an absorption frequency of 350 nm and an extinction frequency of 461 nm). Samples were transferred to Hoechst working solution (at a concentration of 5 μg/ml for 10 min in absolute darkness). The slices were then washed in PBS puffer three times, transferred to Superfrost Plus® slides (Thermo Scientific), covered with Vectashield® Mounting Medium (Vector Laboratories LTD, Peterborough, UK) and Menzel coverslips (Gerhard Menzel GmbH, Braunschweig, Germany). Microscopy was performed with an Olympus BX51 fluorescence microscope with an Olympus DP71 camera using a cell sens standard program. A grey-scale photo was taken and the histogram adjusted to 10–125. False color images were obtained in green and red for further examination. Two representative regions were selected for each specimen and two specimens per mouse were evaluated at an enlargement of 200 × (about 150 enterocytes/field). The apoptotic cell ratio was determined as percentage of apoptotic nuclei in relation to the total number of enterocytes.

### Tight junction and apoptosis protein RNA gene expression

Quantitative real time PCR for gut permeability was performed in ileum sections. Markers (tight junction protein 1, occludin-1, claudin 2 and claudin 4) were chosen according to previous reports in the literature^18^. Total RNA from frozen mouse ileum segments was isolated with a Qiagen miRNeasy Micro Kit (Qiagen, Hilden, Germany) with DNAse treatment (Qiagen, Hilden, Germany) according to manufacturer’s instructions. RNA yield was quantified on a NanoDrop 2000c spectrophotometer. For reverse transcription 1 µg of total RNA was used in the High Capacity cDNA Reverse Transcription Kit (ThermoFisher Scientific) according to manufacturer’s instructions.

The cDNA was used as template for quantitative RT-PCR reactions in a BioRad CFX 384 real-time PCR detection system with the assays β-actin (Actb; Mm00607939_s1), hydroxymethylbilane (Hmbs; Mm01143545_m1) as housekeeping genes. Tight-junction-protein-1 (TJP-1; Mm00493699_m1), occludin-1 (OCLDN-1; Mm00500912_m1), claudin 4 (CLDN-4; Mm00515514_s1) and claudin 2 (CLDN-2; Mm00516703_s1) were selected for tight junction evaluation. Additionally, Bax (Bax; Mm00432051_m1), Bad (Bad; Mm00432042_m1), Caspase 3 (Casp3; Mm0195085_m1), Lamin B1 (Lmnb1; Mm0521949_m1), Bak1 (Bak1; Mm00432045_m1) and Bcl2 (Bcl2; Mm00477631_m1) were chosen for characterization of apoptosis (ThermoFisher Scientific).

Briefly, in 10 µl reactions 4 µl cDNA were used in triplicates in a PCR reaction with 5 µl TaqMan Genexpression MasterMix (ThermoFisher Scientific), 0.5 µl assay and 0.5 µl dH_2_O. Cycling conditions were of initial UDG incubation at 50 °C for 2 min, enzyme activation at 95 °C for 10 s followed by 40 cycles of denaturation at 95 °C for 15 s and annealing and extension at 60 °C for one minute. β-actin and Hmbs genes were used as housekeeping genes for normalization.

### Tight junction protein ELISA

Additionally to the PCR, an ELISA was conducted for claudin 2 (ABIN1745307; antikörperonline.com), claudin 4 (ABIN1745294; antikörperonline.com) and occludin-1 (ABIN773507; antikörperonline.com) of ileum samples. All measurements were conducted from tissue homogenates according to the manufacturer’s instructions.

### Statistics

Data was collected in Microsoft Excel 2016® spreadsheets. For statistical analysis data was transferred to SPSS 22.0®. Graphical imaging was performed with GraphPad Prism 7®. Nominal and ordinal data are displayed in numbers and percent, metric data as median and interquartile range (IQR). Comparisons of independent groups were conducted with Mann–Whitney-U-Tests. A Pearson test was performed to search for correlations between different parameters. P values < 0.05 were regarded as statistically significant differences.

### Ethics approval

All described methods were carried out in accordance with relevant guidelines and regulations and were approved by the veterinary board (Austrian Federal Ministry of Education, Science and Research, BMWFW 66.010/0028-WF/V/3B/2017).

## Results

**Figure 1:**
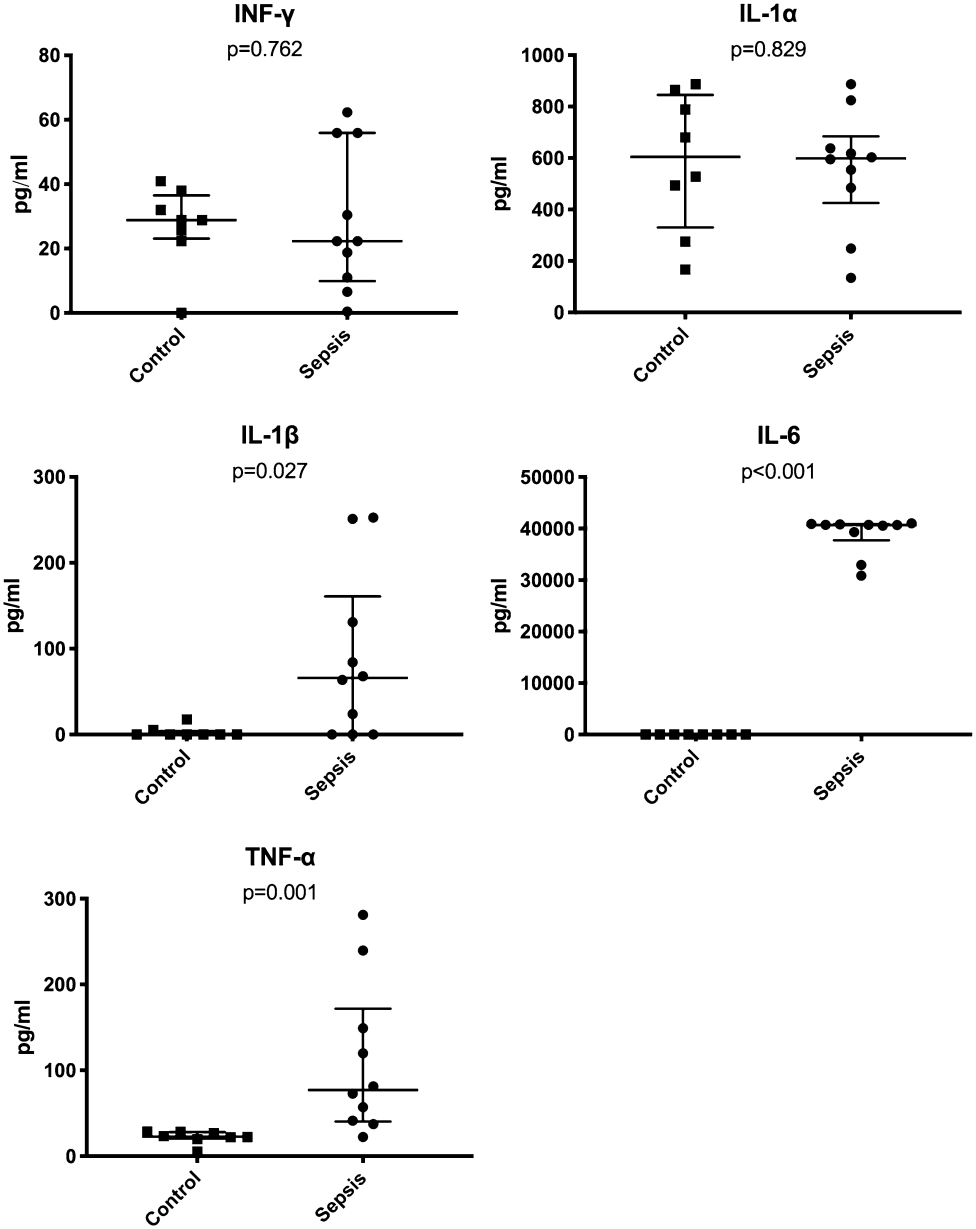
Inflammatory cytokine serum levels in control and sepsis animals.

The clinical sepsis scores of all animals are displayed in Supplement 1. Sepsis caused significantly increased serum levels of IL-1β (control median 2.9, IQR 4.0 vs. sepsis median 65.8, IQR 161.0; p = 0.027), IL-6 (control median 12.4, IQR 9.6 vs. sepsis median 40,704.8, IQR 3,130.3; p < 0.001) and TNF-α (control median 23.0, IQR 7.5 vs. sepsis median 77.1, IQR 131.5; p = 0.001) (Fig. 1). INF-γ and IL-1α were not changed. However, septic animals had significantly increased gut permeability for FITC-dextran (control median 1.2, IQR 0.7 vs. sepsis median18.1, IQR 78.0; p < 0.001).

**Figure 2:**
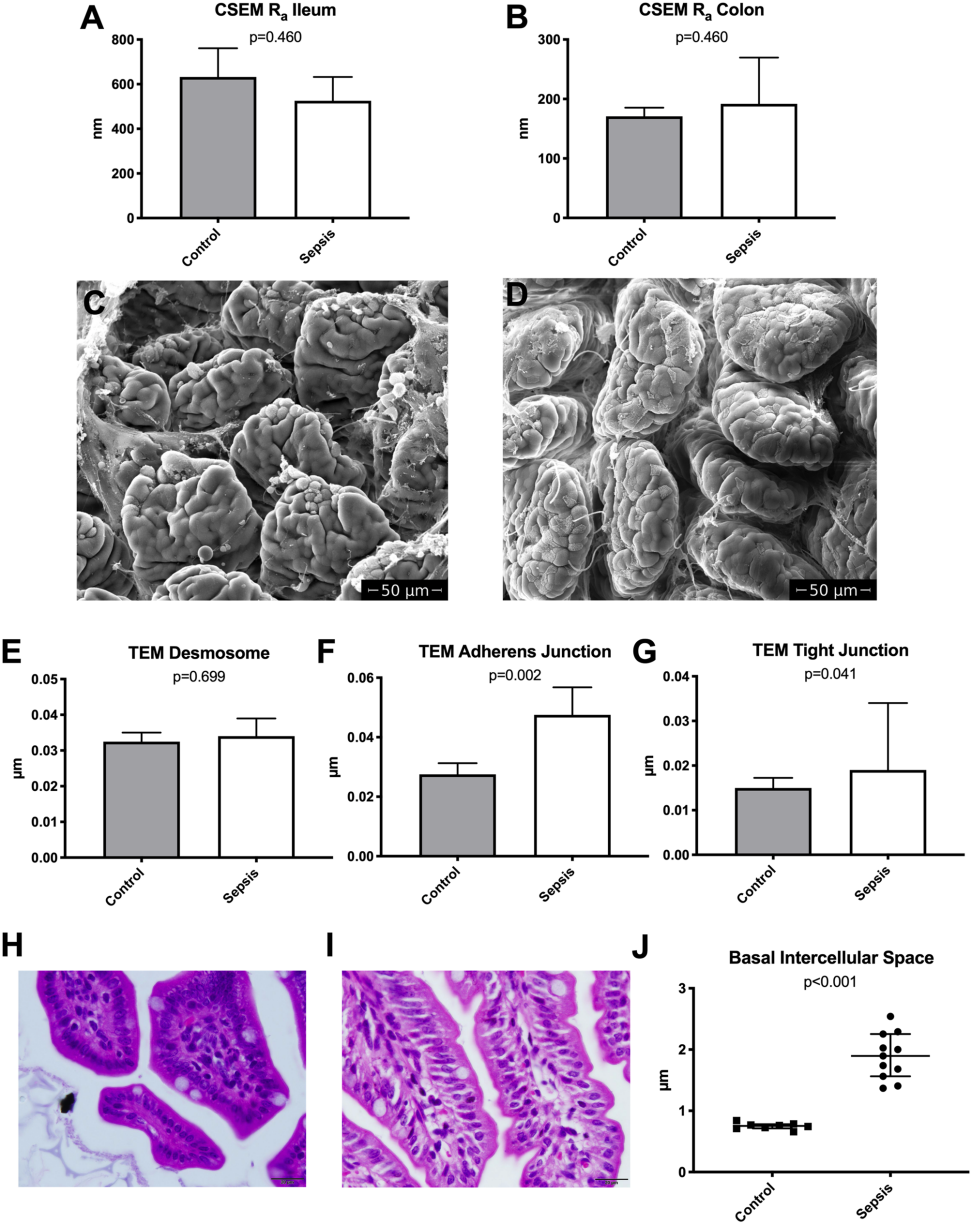
Light and electron microscopy (CSEM and TEM) results. (**A**, **B**) 3D reconstruction-derived surface roughness (R_a_) in ileum (**A**) and colon (**B**) (bar represents median, whiskers IQR). CSEM images of small intestine of a septic (**C**) and a control (**D**) mouse. Intercellular distances at Desmosome (**E**), AJ (**F**) and TJ level (**G**) (bar represents median, whiskers IQR). Bottom row: light microscopy of a control (**H**) and a septic (**I**) mouse (H&E staining, magnification × 400, scale bars represent 20 µm). Septic animals showed significantly increased intercellular distances at the basal levels of the IECs (**J**).

Light microscopy revealed significantly increased intercellular spaces at the basal levels of IECs in septic animals (Fig. 2).

Conventional scanning electron microscopy did not show any evident differences between the two groups in both ileum and colon samples (Fig. 2). There was no statistically significant difference in the surface roughness (R_a_) values comparing the two groups (Fig. 2). A detailed analysis of the TEM images regarding tight junctions, adherens junctions and desmosomes revealed significantly increased intercellular distances at the level of tight and adherens junctions but not in the area of desmosomes (Fig. 2). Median microvillus length was 1.21 µm (IQR 24) in the control group and 1.21 µm (IQR 20) in the sepsis group (p = 0.583).

**Figure 3:**
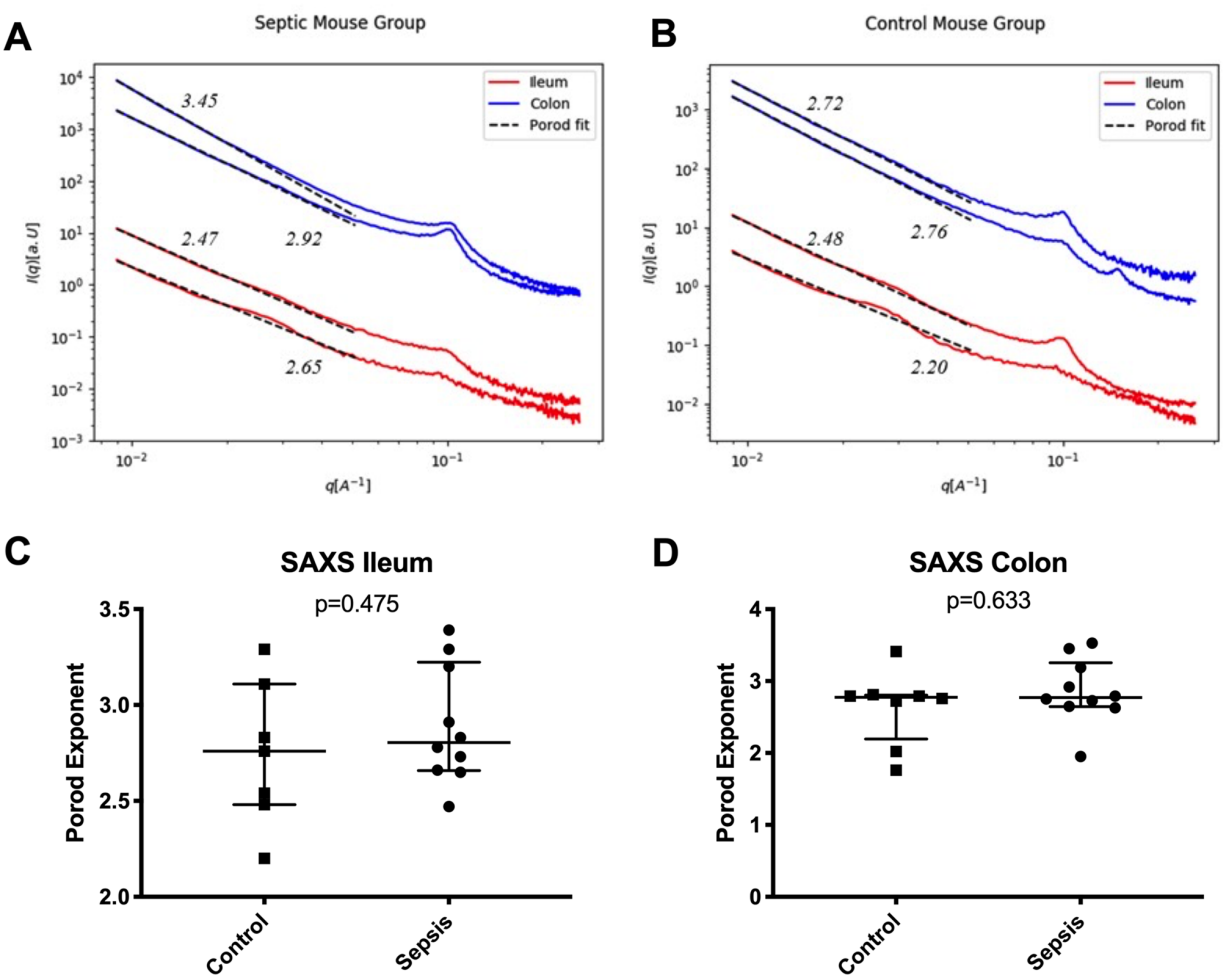
Small-angle X-ray scattering (SAXS). q versus intensity curves of ileum and colon of a septic (**A**) and a control mouse (**B**). Porod exponents of ileum (**C**) and colon (**D**) did not show significant differences.

Figure 3 depicts the results of SAXS measurements without statistically significant difference of the Porod exponents of the two groups.

**Figure 4:**
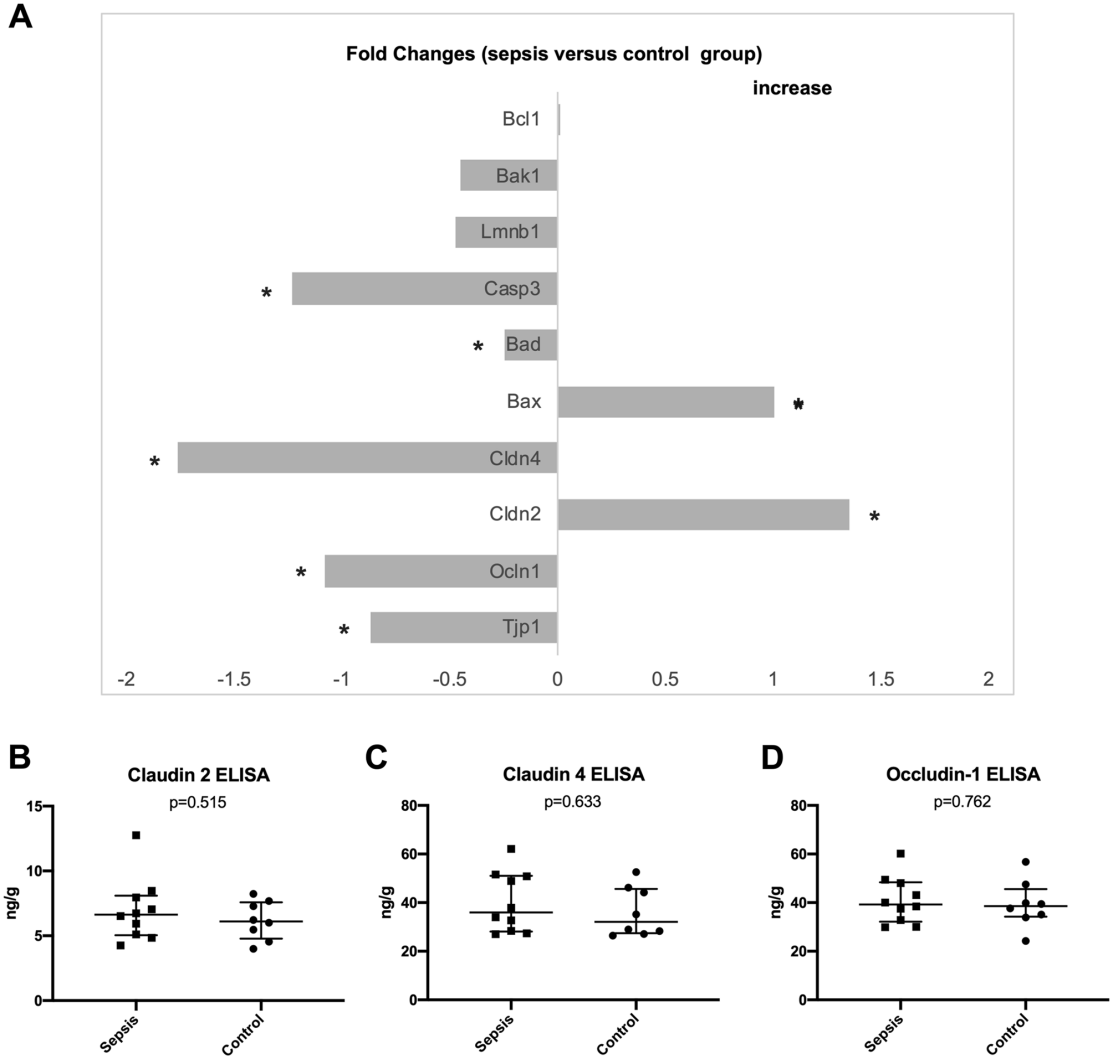
Tight junction and apoptosis protein RNA expression in fold-changes (sepsis versus control) normalized for housekeeping genes as determined by PCR analysis (**A**); an asterisk marks parameters with significant differences between sepsis and control group. ELISA for tight junction proteins (**B**–**D**).

Tight junction protein gene expression analyses of ileal samples revealed that CLP caused a significantly increased expression, i.e. lower corrected Ct values, of CLDN-2 and decreased expression (higher corrected Ct values) of CLDN-4, TJP-1 and OCLN-1 (**Fig. ****4**** and **Supplement 3). However, ELISA measurements of ileum samples could not confirm these findings on the protein level (no significant differences for claudin 2, claudin 4 and occludin-1).

The apoptosis rate assessed by HOECHST staining was not significantly different between the two groups (median 2.8, IQR 3.1 in the control group vs. median 1.8, IQR 3.2 in the sepsis group; p = 0.573). Apoptosis gene expression showed significant decreases of Bad and Caspase 3 expression and significant increases of Bax expression in septic animals (Fig. 4).

Conventional light microscopy of H&E stains showed significantly increased modified Marsh Oberhuber scores for bowel wall inflammation in ileum (control median 0.1, IQR 0.4 vs. sepsis median 1.3, IQR 0.8; p = 0.003) and colon (control median 0.1, IQR 0.4 vs. sepsis median 1.9, IQR 0.6; p < 0.001) samples. There was no statistically significant difference for villus height (VH), crypt depth (CD) or VH:CD ratio in ileum and colon samples. H&E staining of ileum samples also showed significantly increased intercellular distances at the basal level of IECs (Fig. 2).

### Correlation analysis

Correlation analysis showed a statistically significant correlation between FITC-dextran levels and the cytokines IL-6 (coefficient 0.520; p = 0.027) and TNF-α (coefficient 0.635; p = 0.005, as well as the gene expression of OCLN-1 (coefficient 0.649; p = 0.004), the intercellular distance at the TJ level (coefficient 0.679; p = 0.015) but not with R_a_ or the Porod exponent.

## Discussion

The present study investigated the influence of CLP-induced sepsis on different parameters characterizing the integrity of bowel wall of ileum and colon. Cecal ligation and puncture as applied in this study is a well-established method to induce polymicrobial abdominal sepsis^17,20,21^ which was confirmed by significantly increased serum levels of the pro-inflammatory cytokines IL-1β, IL-6 and TNF-α eight hours after CLP.

Sepsis is associated with disruptions of the intestinal barrier function leading to increased bowel permeability^5,21^. We were able to show this disruption by the increased levels of serum FITC-dextrane in the septic animals. The time points between FITC-dextran gavage, CLP and euthanasia were chosen according to other reports in the literature aiming to investigate the early phase of sepsis^21^.

A variety of different mechanisms can be hypothesized as the underlying reasons for intestinal barrier dysfunction during sepsis including epithelial apoptosis, alterations of tight junction composition^6,21^ and alterations of the nanoarchitecture of the bowel wall. In previous reports, increased intestinal epithelial apoptosis and villus atrophy were found to be responsible for increased FITC-dextran permeability in septic mice after 24, 48 and 72 h^20^. In contrary, HOECHST staining in our experiments revealed no significant increase in intestinal epithelial cell apoptosis in ileum samples. Apoptosis gene expression showed divergent results. The expression of pro-apoptotic Bax was significantly increased in septic animals. At the same time, the expression of pro-apoptotic Bad and Caspase 3 was significantly decreased in these animals. However, the corrected Ct values showed fold changes below 1.5 for all genes investigated; thus, the PCR findings were significant for some genes but the biological relevance can be questioned. The relatively shorter time interval between CLP and euthanasia in our experiment as compared to other reports seems to be the most likely reason for the low apoptosis rate. Similarly, conventional histology could not demonstrate reduced villus height or crypt depth as a known sign of apoptosis in ileum and colon of septic animals.

Another possible mechanism for intestinal hyperpermeability are alterations of tight junctions with increased porosity in septic animals^20,21^. In this regard, we could demonstrate significant increases of the expression of claudin-2 (CLDN-2) paired with decreased expression of tight junction protein 1 (TJP-1), occludin-1 (OCLN-1) and claudin-4 (CLDN-4) in ileum samples of septic mice. Regarding the fold changes only claudin-4 reached levels higher than 1.5 (compare Fig. 4). These findings were in parallel with significantly increased bowel wall inflammation as characterized by increased modified Marsh Oberhuber scores in ileum and colon samples following CLP and increased width of TJ and AJ of ileal samples in septic animals compared to control animals (compare Fig. 2).

Alterations of the cellular ultrastructure may represent a further reason for intestinal hyperpermeability associated with sepsis. However, data concerning ultrastructural analysis of bowel samples in sepsis are lacking in the literature. There is only a small number of studies applying small-angle X-ray scattering (SAXS) for ultrastructural analysis of organic tissues. Past experiments performed in soft tissue have mainly focused on breast tissue^15,16,22^. SAXS, however, has not yet been used to experimentally assess the ultrastructure of the bowel wall during sepsis. In the present study we were not able to find alterations of the cellular ultrastructure between control and septic animals as assessed by SAXS. Unlike microscopy techniques, where local and unique features are depicted, SAXS yields averaged nanostructural parameters of the sample volume irradiated by the beam. Therefore, it is particularly useful for investigating nanostructural features that occur in the irradiated volume in great quantity, because each nanoscale object in the beam contributes to yield an overall detectable scattering signal. Furthermore, the strength of the method lies in the non-destructive nature and the possibility to use it on hydrated soft tissue. In the case of hyperpermeable bowels associated with sepsis, a great amount of nanosized pores of defined size should yield a distinct shoulder in the SAXS curves in the q range between 0.01 and 0.05 A^−1^. This is, however, not visible in our results. Alternatively, a large distribution of pore sizes could make the shoulder vanish (signal smearing out), but should still be visible in a change of fractal dimension as determined from power law fitting. We therefore suggest that the intestinal hyperpermeability in the early phase of sepsis is not associated with the formation of great numbers and high density of nanosized pores.

Likewise, CSEM which we have used to examine bowel samples in septic mice could not reveal structural changes. CSEM was used to analyze the bowel surface choosing surface roughness as parameter for group comparison. We could not determine alterations of the surface roughness between the groups 8 h after induction of sepsis. An analysis of the intercellular contacts showed increased intercellular spaces at the basal levels of IECs of septic animals in H&E stainings. While on the first view there seemed to be no alterations of the intercellular contacts (specially at the TJ level) more detailed measurements of intercellular contacts showed significantly dilated intercellular spaces of septic animals at the level of adherens and tight junctions in TEM images. Neither conventional histology nor TEM could demonstrate significant differences of the microvilli length between septic and control mice.

Limitations of the present study include that we have only assessed the early phase of sepsis 8 h following CLP. Nevertheless, previous studies have shown that while intestinal permeability is elevated 6 to 48 h after the onset of sepsis it reaches a peak between 6 and 12 h following induction of sepsis^21^. Moreover, we cannot rule out regional heterogeneity of the different bowel sections regarding the parameters assessed at microscopy.

In conclusion, the present study highlights that bowel wall hyperpermeability in the early phase of experimental polymicrobial sepsis is most likely caused by alterations of the intercellular contacts (AJ and TJ) and not by apoptosis or increases in size or number of nanopores of intestinal epithelial cells.

## Supplementary information

Supplementary Information 1 (PDF 1250 kb)

## Data Availability

The datasets generated during and/or analyzed during the current study are available from the corresponding author on reasonable request.

## References

[1] Gaieski D, Edwards J, Kallan M, Carr BG (2013). Benchmarking the incidence and mortality of severe sepsis in the United States. Crit. Care. Med..

[2] Tisdale MJ (1997). Cancer cachexia: Metabolic alterations and clinical manifestations. Nutrition.

[3] Blikslager AT, Moeser AJ, Gookin JL, Jones SL, Odle J (2007). Restoration of barrier function in injured intestinal mucosa. Physiol. Rev..

[4] Podolsky DK (1999). Mucosal immunity and inflammation. V. Innate mechanisms of mucosal defense and repair: The best offense is a good defense. Am. J. Physiol..

[5] Al-Sadi R, Boivin M, Ma T (2009). Mechanism of cytokine modulation of epithelial tight junction barrier. Front. Biosci. (Landmark Ed.).

[6] Zeissig S (2007). Changes in expression and distribution of claudin 2, 5 and 8 lead to discontinuous tight junctions and barrier dysfunction in active Crohn's disease. Gut.

[7] Kawano M, Miyoshi M, Ogawa A, Sakai F, Kadooka Y (2016). Lactobacillus gasseri SBT2055 inhibits adipose tissue inflammation and intestinal permeability in mice fed a high-fat diet. J. Nutrition. Sci..

[8] Tong LC (2016). Propionate ameliorates dextran sodium sulfate-induced colitis by improving intestinal barrier function and reducing inflammation and oxidative stress. Front. Pharmacol..

[9] Sui T (2014). In situ X-ray scattering evaluation of heat-induced uiltrastructural changes in dental tissues and synthetic hydroxyapatite. J. R. Soc. Interface.

[10] Di Cola E, Grillo I, Sristori S (2016). Small angle X-ray and neuron scattering: Powerful tools for studying the structure of drug loaded liposomes. Pharmaceutics.

[11] Fratzl P (1994). Statistical model of the habit and arrangement of mineral crystals in the collagen of bone. J. Stat. Phys..

[12] Lichtenegger H, Reiterer A, Stranzl-Tschegg S, Fratzl P (1999). Variation of cellulose microfibril angles in softwoods and hardwoods—A possible strategy of mechanical optimization. J. Struct. Biol/.

[13] Pabisch S, Awagermaier W, Zander T, Li C, Fratzl P (2013). Imaging the nanostructure of bone and dentin through small- and wide-angle X-ray scattering. Methods Enzymol..

[14] Poundarik A, Boskey A, Gunberg C, Svashishth D (2018). Biomolecular regulation, composition and nanoarchitecture of bone mineral. Nature.

[15] Fernández M (2008). USAXS and SAXS from cancer-bearing breast tissue samples. Eur. J. Radiol..

[16] Sidhu S (2011). Classification of breast tissue using a laboratory system for small-angle X-ray scattering (SAXS). Phys. Med. Biol..

[17] Short SS (2013). Low doses of celecoxib attenuate gut barrier failure during experimental peritonitis. Lab. Investig. J. Tech. Methods Pathol..

[18] Buchheister S (2017). CD14 plays a protective role in experimental inflammatory bowel disease by enhancing intestinal barrier function. Am. J. Pathol..

[19] Adelman D (2018). Measuring change in small intestinal histology in patients with celiac disease. Am. J. Gastroenterol..

[20] Dominguez J (2013). Inhibition of IKKb in enterocytes exacerbates sepsis-induced intestinal injury and wordsens mortality. Crit. Care Med..

[21] Yoseph B (2016). Mechanisms of intestinal barrier dysfunction in sepsis. Shock.

[22] Conceicao A, Antoniassi M, Poletti M (2009). Analysis of breast cancer by small angle X-ray scattering (SAXS). Analyst.

