# Supplementary material for: Examination of intestinal ultrastructure, bowel wall apoptosis and tight junctions in the early phase of sepsis
Source: Sci Rep. 2020 Jul 13;10:11507. doi: 10.1038/s41598-020-68109-9 (PMC7359326; doi:10.1038/s41598-020-68109-9)
Supplement: Supplementary file 1 — Supplementary Information 1 (PDF 1250 kb) [file 41598_2020_68109_MOESM1_ESM.pdf]

Examination of Intestinal Ultrastructure, Bowel Wall Apoptosis and Tight Junctions in the Early Phase of Sepsis

Beate Obermüller[1, 3], Noemi Frisina[2], Martin Meischel[2], Georg Singer[3], Stefanie Stanzl-Tschegg[2], Helga Lichtenegger[2], Dagmar Kolb[4], Ingeborg Klymiuk[5], Holger Till[3] and Christoph Castellani[3]

[1] Department of Biomedical Research, Medical University of Graz, Austria

[2] Department of Physics and Materials Science, University of Natural Resources and Life Sciences, Vienna, Austria

[3] Department of Paediatric and Adolescent Surgery, Medical University of Graz, Austria

[4] Core Facility Ultrastructure Analysis, Center for Medical Research, Gottfried Schatz Research Center, Medical University of Graz, Austria

[5] Core Facility Molecular Biology, Center of Medical Research, Medical University of Graz, Austria

Supplement 1: Clinical Sepsis Score of both groups and applied scoring system

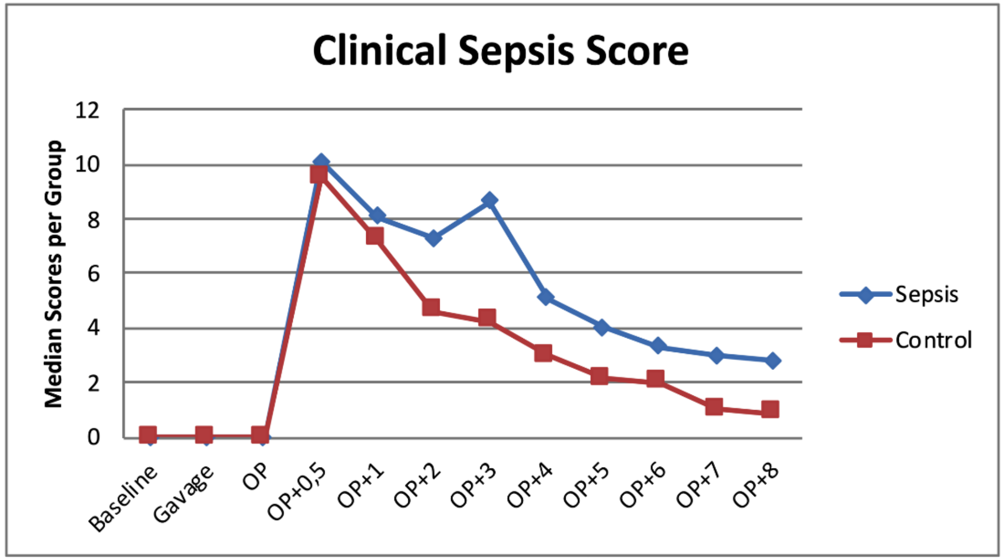

BioMed RESEARCH GRAZ

Determination of humane endpoint criteria in animal experiments

Observation	Score
**I Body weight**	
-unaffected or weight gain	0
-alteration < 5%	1
-Weight loss 5-10%	5
-Weight loss 11-20%	10
-Weight loss >20%	20
**II General health condition**	
-smooth and shiny fur; clean orifices; clear and shiny eyes	0
-change of the fur (reduce or excessive body care)	1
-rough hair coat, unkempt appearance, neglected orifices, abnormal skin surface (small wounds), cloudy eyes, discharge form eyes, starting/minor rectal prolapse, abnormal posture, increased muscle tone	5
-rough and dirty hair coat, humid orifices (stuck together), moderate rectal prolapse, dehydration, abnormal posture, cloudy eyes, excessive muscle tone	10
-cramps, paralysis (trunk muscles, extremities); breath sounds; severe rectal prolapse	20
**III Behavior**	
-normal behaviour (sleeping, the animal shows a reaction to blowing and touch, curiosity, social interaction)	0
-slight deviations of the normal behaviour	1
-unusual behaviour, limited motor activity or hyperkinetic	5
-isolation, lethargy; excessive hyperkinetic or behavioural stereotypy; disorder of coordination	10
-Pain sounds while seizing; Self-induced trauma (autoaggression)	20
**IV Clinical findings**	
-normal temperature an respiration, warm extremities, Mucous membranes are well supplied with blood, normal abdomen	0
-slight deviations of the normal situation	1
-the animal feels colder as normal, cold extremities, pale mucous membranes	5
-moderate deviation of the temperature, respiration + or – 30%, enlarged abdomen	10
-high deviations of the temperature, respiration + or – 50%, blood in urine of feces, diameter of a pressable tumour > 2com (in rodents)	20
**V Study specific humane endpoints**	
OP wound closed, skin around normal	0
OP wound closed, skin around reddish	1
OP wound slightly opened at one point in the upper layer, skin around red	5
OP wound slightly opened at more than one point in the upper layer, skin around red with starting grey boundary of the wound	10
OP wound opened at one point in both layers or completely in the upper layer, skin around red with starting gangrene	20

Evaluation, Measures	Sum of scores
Stress level 0= no stress	0
Stress level 1= slight level of stress, note in PyRat, to be observed carefully	1-9
Stress level 2= moderate levels of stress; note in PyRat, the veterinarian and the project director must be informed, if applicable veterinary care must be initiated	10-19
-Stress level 3= high levels of stress; note in PyRat, with duration the veterinarian and the project director must be informed, veterinary care must be initiated, if applicable the animal must be euthanized or rather the study must be terminated	20 or more

Kontakt: Mag. Beate Obermüller
Letzte Änderung: 12.03.2018

Supplement 2: Width of the intercellular spaces in HE stains (left) and measurement of the intercellular contacts in TEM images (right)

Measurement of intercellular distance in H&E stained sections of ileum samples (100x oil immersion objective lens)

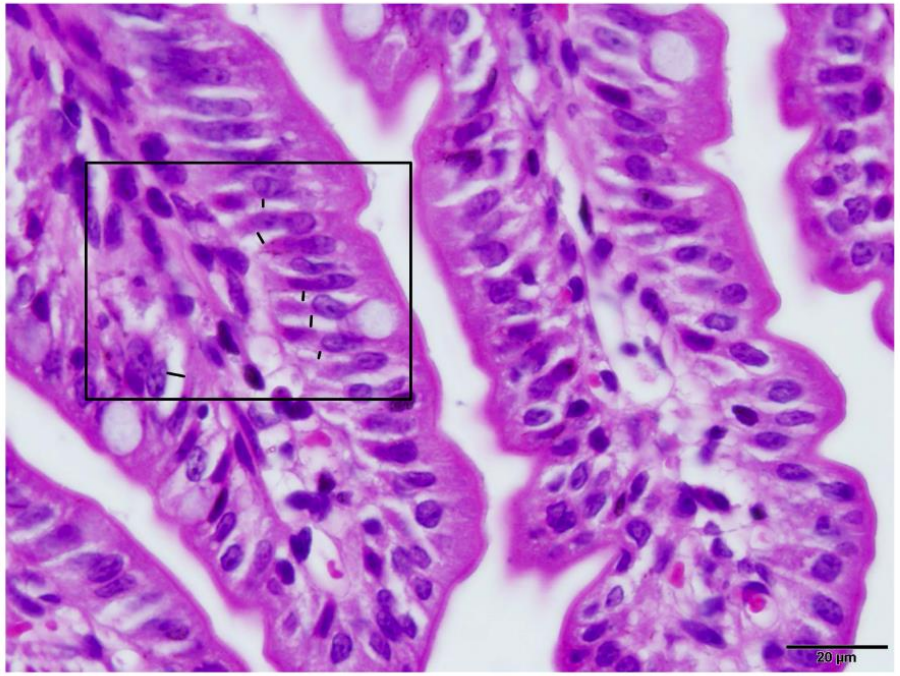

Measurement of intercellular distances at the tight junction (TJ), adherens junction (AJ) and Desmosome (Des)

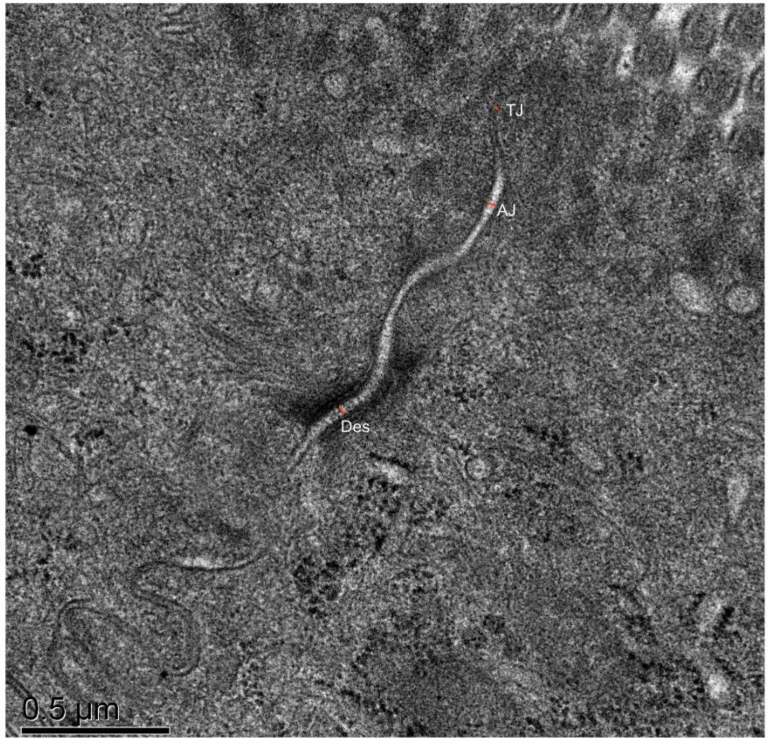

Supplement 3: Ct values corrected for housekeeping genes in qRT-PCR

a.) Tight junction analysis of ileum samples (data displayed as median (IQR)).

	**Sepsis**	**Control**	**p-value**
**TJP**	1.78 (0.20)	1.67 (0.19)	**0.043**
**Ocln**	1.57 (0.49)	1.30 (0.08)	**0.009**
**CLDN-2**	2.99 (0.45)	3.41 (0.23)	**0.001**
**CLDN-4**	-0.20 (0.77)	-0.98 (0.34)	**0.002**

TJP…tight junction protein; Ocln…Occludin, CLDN-2…claudin-2, CLDN-4…claudin-4

b.) Bowel wall apoptosis of ileum samples (data displayed as median (IQR)).

	**Sepsis**	**Control**	**p-value**
**Bax**	5.00 (0.30)	5.36 (0.43)	**0.016**
**Bad**	5.78 (0.57)	5.14 (0.26)	**0.002**
**Casp3**	7.00 (0.22)	6.76 (0.29)	**0.001**
**LamB1**	6.43 (0.25)	6.30 (0.18)	0.122
**Bak**	2.66 (0.18)	2.64 (0.01)	0.237
**Bcl2**	7.43 (0.56)	7.48 (0.31)	1.0

Casp3…Caspase 3, LamB1…Lamin B1
